# Introduction: advanced intelligent computing theories and their applications in bioinformatics

**DOI:** 10.1186/1471-2105-13-S7-I1

**Published:** 2012-05-08

**Authors:** M Michael Gromiha, De-Shuang Huang

**Affiliations:** 1 Department of Biotechnology, Indian Institute of Technology Madras, Chennai 600036, Tamilnadu, India; 2 Machine Learning and Systems Biology Laboratory, Tongji University, 4800 Caoan Road, Shanghai 201804, China

## 

The advancement of techniques in computer science and information technology witnessed the rapid growth of bioinformatics in various diverse areas such as sequence alignment, structure prediction, structure-function relationship, protein interactions, genome annotation, gene expression, microarray data analysis and so on. It is necessary and pertinent to discuss the issues on these topics and analyze the latest developments. The International Conference on Intelligent Computing (ICIC) provided a forum for discussing the recent investigations on bioinformatics related problems using high performance computing and efficient algorithms. Among the 832 submissions 33.7% were selected for presentations at ICIC 2011. Based on the novelty of the manuscripts, presentations and originality only 12 papers were selected as 'high quality', and the extended versions of them are included in the supplement.

The supplement is broadly classified into five categories, structure-function relationship of proteins, protein-protein interactions, gene expression/interaction networks, microarray data analysis and visualization tools. The opening article by Gromiha et al. [1] related various physical, chemical, energetic and conformational properties of amino acid residues with the change of half maximal effective concentration (EC50) due to amino acid substitutions in olfactory receptors. Further, they utilized machine learning methods for discriminating the mutants, which enhance or reduce EC50 values upon mutation. Wang et al. [2] proposed a protein-protein dissimilarity learning algorithm for comparing protein structures using the contextual information of proteins.

Lei et al. [3] developed a robust computational technique for assessing the reliability of protein-protein interactions and predicting the interacting pairs of proteins by integrating manifold embedding with various features. Wang et al. [4] described an algorithm for identifying overlapping modules in protein-protein interaction networks. Cui et al. [5] built a support vector machine model for predicting human proteins that interact with virus proteins and specifically human papillomavirus and hepatitis C virus. Liu et al. [6] constructed an integrated map of protein interaction network in Mycobacterium tuberculosis using machine learning and ortholog-based methods.

Wang et al. [7] presented a network biology approach for investigating drug combinations and their target proteins in the context of genetic interaction networks and related human pathways with an aim to understand the underlying rules of effective drug combinations. Hsiao et al. [8] proposed an incremental evolutionary approach using network robustness for inferring gene regulatory networks with an application to deal with a large number of network parameters. Bevilacqua et al. [9] explored the issue of microarray data merging and used distant metastasis prediction for classifying three different sets of breast cancer data. Park et al. [10] analyzed the whole brain microarray data and physical connectivity of hippocampus with other brain regions to identify the genes related to Alzheimer's disease and their interactions with proteins. Ayadi et al. [11] described a stochastic pattern-driven neighborhood search algorithm for biclustering microarray data. In the last article, Jung et al. [12] described the development of a JAVA based stand-alone program for detecting and visualizing of genomic variants, which enables the manual exclusion of erroneous signals. It is also capable of visualizing genomic data from different sources such as data from comparative genomic hybridization arrays and sequence alignment format files.

The guest editors of the supplement would like to thank the Executive Editor of *BMC Bioinformatics *Professor Kate Rice for providing an opportunity to publish some of the excellent papers presented in ICIC 2011. We also wish to thank Ms. Isobel Peters and Ms. Catherine Wells for their help and support in editing the supplement. Finally, our sincere thanks to all the authors of the papers selected for publication in this issue. This work was partially supported by the grants of the National Science Foundation of China, Nos. 61133010, & 31071168.

## Competing interests

The authors declare that they have no competing interests.
